# Analysis of m7G-related signatures in the tumor immune microenvironment and identification of clinical prognostic regulators in ovarian cancer

**DOI:** 10.3389/fimmu.2025.1595618

**Published:** 2025-08-14

**Authors:** Kunyu Wang, You Wu, Miao Ao, Wei Mao, Haixia Luo, Yan Song, Bin Li

**Affiliations:** ^1^ Department of Gynecology Oncology, National Cancer Center/National Clinical Research Center for Cancer/Cancer Hospital, Chinese Academy of Medical Sciences and Peking Union Medical College, Beijing, China; ^2^ Department of Pathology, National Cancer Center/National Clinical Research Center for Cancer/Cancer Hospital, Chinese Academy of Medical Sciences and Peking Union Medical College, Beijing, China

**Keywords:** m7G, immune microenvironment, NUDT16, ovarian cancer, DCP2

## Abstract

Ovarian cancer (OV) is the most lethal gynecological malignancy in the world. At present, the effect of m7G modification-related genes on the development of ovarian cancer remains unclear. We performed consensus clustering of ovarian cancer samples based on the expression of 24 m7G modification-related genes, and obtained 2 subtypes. There were some differences in immune cell infiltration between the two subtypes. Furthermore, enrichment analysis showed that differential genes were mainly enriched in several pathways and biological processes, including positive translation regulation and TRAPP complex. Multivariate cox regression analysis confirmed two genes (DCP2 and NUDT16) related to prognosis for the construction of risk score prediction models. The risk map of survival status showed that the high-risk samples had a shorter survival time (p<0.05). Risk score was an independent prognostic factor for OV and correlated with immunotherapy response. We also performed network analysis for DCP2 and NUDT16. We further explored the effects of the genes on cellular function and prognosis. In conclusion, this study provided a new perspective for the development mechanism of ovarian cancer.

## 1 Introduction

Ovarian cancer (OV) is one of the three most common malignant tumors in women, and the mortality rate has always been the first in gynecological malignant tumors, which seriously threatens women’s health (1, 2). Epithelial ovarian cancer is the leading cause of death from gynecologic cancers in the United States, with less than half of patients living more than 5 years after diagnosis (3). Additional risk factors were high BMI and occupational risks for ovarian cancer (4). At present, there is an urgent need to deeply study the pathogenesis of ovarian cancer in order to generate new effective therapeutic targets and therapies.

In the comprehensive treatment of ovarian cancer, platinum drugs are the first-line drugs for combined therapy after cytoreductive surgery, and are currently the standard treatment for ovarian cancer, but the effect of prolonging the survival of patients is still not very obvious (5, 6). Because tumor tissues become resistant to cisplatin (7), and the response rate to cisplatin therapy decreases especially at the time of recurrence (8). Bevaczumab and poly-ADP ribose polymerase inhibitors (PARPi) now play a maintenance role in first-line or platinum-sensitive relapse treatment of ovarian cancer (9, 10). In the tumor immune microenvironment, TILs are major components of tumor-infiltrating immune cells, including T cells, B cells, and NK cells, and have been reported to affect cancer progression and response to immunotherapy (11). The heterogeneity of tumor immune microenvironment is an important factor affecting the response of immunotherapy (12). The emergence of tumor immunotherapy has brought a new dawn to ovarian cancer patients. However, the current response rate of immunotherapy is low, and the application effect in ovarian cancer treatment is limited. Therefore, it is of great clinical significance to study the regulation factors of the immune microenvironment of ovarian cancer and promote the personalized immunotherapy of ovarian cancer.

Several researches have indicated that RNA epigenetic modifications can affect the progression and metastatic spread of ovarian cancer, potentially serving as promising targets for cancer therapy (13, 14). RNA modification is a dynamic and reversible process regulated by a series of writers, erasers and readers (15). Among epigenetics, N(7)-methylguanosine (m(7)G), as the epigenetic modification at the 5’ cap of mRNA, plays essential roles in regulating mRNA translation and splicing (16, 17). This essential cap modification stabilizes transcripts, prevents exonucleolytic degradation, and regulates nearly every stage of the mRNA life cycle, including transcription elongation, pre-mRNA splicing, polyadenylation, nuclear export, and translation. In addition to being part of the cap structure, m7G is also present inside tRNA and rRNA (18, 19), and internal m7G modifications affect RNA processing and function and are thought to be involved in human diseases, including tumors (20). Previous studies have predicted potential m7G locus associated with ovarian cancer from a high-throughput computational perspective, and obtained correlation conclusions (21). A centralized resource m7GHub V2.0 that supports the sharing, annotation, and customized analysis of m7G data will significantly facilitate m7G research across diverse physiological contexts (22). RMBase v3.0 also enabled integrated analysis of diverse RNA modification profiles (23). A notable advancement in RMVar 2.0 is the combination of allele-specific RNA modification analysis to identify RNA modification-associated variants (24). An important well-studied regulator of m7G in mammals is methyltransferase-like 1 (METTL1), which binds to its corresponding cofactor WD repeat domain 4 (WDR4) to install m7G modifications in tRNA, miRNA, and mRNA (25, 26) while METTL1 is associated with favorable survival in patients with ovarian serous cystad enocarcinoma (27). In ovarian cancer, METTL1-mediated tRNA m7G modification enhances the translation of AKT/mTOR pathway-associated proteins and promotes cell growth and metabolic reprogramming. Specifically, m7G-modified tRNAs preferentially decode AGA codon-rich mRNAs (e.g., AKT1, mTOR), thereby enhancing the expression levels of these oncogenes (26). METTL1 promotes the processing and maturation of miR-21 precursor through m7G modification of specific tRNAs, which in turn inhibits the expression of its target genes (e.g., PTEN), activates the PI3K/AKT pathway, and ultimately leads to chemoresistance (28). In addition, previous research has reviewed m7G’s role in tumor immunity, emphasizing its impact on T cell exhaustion and macrophage M2 polarization through m7G-dependent mRNA stability (29). However, it is still unclear whether m7G modification affects the immune microenvironment and prognosis of ovarian cancer. We hypothesized that m7G modification plays an important role in the pathogenesis of ovarian cancer, and tried to conduct a series of studies.

In this study, based on the expression of 24 m7G-modifification regulators, we performed consensus clustering on a large number of ovarian cancer samples in the database, and divided the ovarian cancer samples into two subtypes. In the tumor microenvironment, the immune cell composition varies significantly. Enrichment analysis showed that the differential genes between subtypes were mainly enriched in pathways such as positive regulation of translation, TRAPP complex and protease binding. Multivariate cox regression analysis identified 2 genes DCP2 and NUDT16 to build predictive models. Risk scores were significantly associated with prognosis in ovarian cancer. Further analysis showed that 2-m7G regulators as independent OV prognostic factors. Network analysis predicted 9 target transcription factors for DCP2 gene and 4 target transcription factors for NUDT16 gene, suggesting a possible complex functional network of these two genes. In addition, ovarian cancer risk scores correlated with response to immunotherapy. Our study suggests that m7G modifies key regulatory genes that may play an important role in the pathogenesis of ovarian cancer.

## 2 Materials and methods

### 2.1 Datasets

Gene expression profiling data of OV patients were obtained from 2 independent patient cohorts, including TCGA-OV, GSE30161, and only OV samples were retained for further analysis. The TCGA-OV data were annotated with gene names using the GENCODE22 annotation file, and the TCGA-OV patient survival data and clinical data, including survival time, survival status, age, and tumor stage, were obtained from UCSC xena. In the end, 352 samples were included in TCGA-OV, 58 samples were included in GSE30161, TCGA-OV was used for model construction, and GSE30161 was used for model validation.

### 2.2 Consensus clustering of m7G-related genes

M7G-related genes from the existing literature (21) and related gene sets were obtained from GOMF_M7G_5_PPPN_DIPHOSPHATASE_ACTIVITY,GOMF_RNA_CAP_BINDING GOMF_RNA_7_METHYLGUANOSINE_CAP_BINDING. 24 m7G-related genes can be annotated in the final meta dataset:(DCP2, IFIT5, EIF3D, EIF4G3, NSUN2, GEMIN5, AGO2, NUDT10, EIF4E, EIF4E2, NCBP2, NUDT11, NUDT3, NCBP1, METTL1, LARP1, NUDT4, EIF4E3, SNUPN, WDR4, LSM1, NUDT16, DCPS, CYFIP1),Based on the expression profile data of the 24 genes, the ConsensusClusterPlus K-means clustering algorithm was used to cluster TCGA-OV patients to obtain 2 subtypes. The clustering was repeated 1000 times to ensure the accuracy and stability of the results.

### 2.3 Identification of differentially expressed genes between m7G patterns

We used the limma package to identify DEGs between the 2 subtypes, with thresholds set at corrected p<0.05, |logFold Change|>1. In order to display the enriched pathways between different subtypes, we performed GO and KEGG enrichment analysis between each two subtypes, and visualized the top 5 pathways with the most significant enrichment results. Gene set enrichment analysis was also performed between subtypes (c2.cp.kegg.v7.5.1.symbols.gmt and c5.go.v7.5.1.symbols.gmt of the reference gene set MSigDB database), here we take the top 10 Each pathway is visually displayed, and the above enrichment analysis process is completed by the clusterProfiler package.

### 2.4 Comparison of immune cell infiltration among m7G patterns

In order to explore the degree of immune cell infiltration between different subtypes, we used the IOBR package to evaluate the immune cell infiltration of the TCGA-OV data set with the ESTIMATE algorithm, the MCPcounter algorithm and CIBERSORT, and obtained the immune cell infiltration of each sample in the two algorithms. Among them, the indicators evaluated by the ESTIMATE algorithm include immune score, matrix score and tumor purity, the MCP counter algorithm includes 10 kinds of immune cells such as CD8+ T cells and NK cells, and the CIBERSORT algorithm includes 22 kinds of immune cells such as T cells and B cells. Differences in immune cell infiltration between different subtypes were considered significant with a p-value less than 0.05.

### 2.5 Support vector machines and multivariate cox regression and validation of the prognostic m7G signatures

We used the support vector machine algorithm of the e1071 package to select the prognosis-related features on the TCGA-OV expression profiling data of 24 m7G-related genes, and 21 of the 24 genes were screened out and further used the survival package. Multivariate cox analysis was used to construct predictive models and survival analysis was performed using the survival package. At the same time, we validated the model in the GSE30161 dataset, and in a survival analysis p less than 0.05 was considered a significant difference in survival. In addition to this, we integrated risk scores and clinical features for univariate and multivariate cox analyses to validate that risk scores are predictive markers independent of other clinical features, and constructed nomograms using the rms package based on clinical features associated with prognosis, to make a more accurate prediction of patient prognosis, and the results of nomogram prediction are verified by calibration curve, ROC curve and decision curve analysis to ensure that the nomogram is accurate in predicting the 1-year, 3-year and 5-year survival rate of patients.

### 2.6 Construction of regulatory network

First, we use the starBase database to predict potential miRNAs targeting hub genes (take the interactions that also exist in the TargetScan database), and identify the gene-miRNA regulatory network. Then, the online tool of ChEA3 was used to identify gene-transcription factor interaction pairs (the interaction relationship with an interaction score greater than 800),which was used to establish an upstream regulatory network. Additionally, the Comparative Toxicogenomics database was queried for compounds with potential relationships to core genes. Finally, the visualization of the core gene regulatory network is implemented based on the igraph package.

### 2.7 Prediction of immunotherapy response

We obtained the risk score for each sample based on the prediction model in the IMvigor210 and GSE78220 immunotherapy response datasets, performed survival analysis for high and low risk groups, and compared the response to immunotherapy between high and low risk groups.

### 2.8 Statistical analysis

Statistical analysis was performed by R (version 4.1.1). The Wilcoxon rank-sum test was used for comparison between the two groups, and the Kruskal-Wallis test was used for multiple comparisons. The cut-off point for each subgroup was determined by the survminer package in R. Kaplan-Meier curves for OS analysis are presented between different subgroups, followed by log-rank tests. Multivariate Cox regression analysis was used to assess the association between OS, clinicopathological characteristics and risk scores and was presented by the forestplot package. p-values were corrected by Bonferroni. Two-sided p<0.05 was considered statistically significant.

### 2.9 Cell lines and culture

A2780 cells were derived from the National Experimental Cell Resource Sharing Platform (Beijing, China), and 3AO cells were purchased from the Cell Bank of the Chinese Academy of Sciences (Shanghai, China). A2780 cells were cultured in DMEM supplemented with 10% FBS, and 3AO were maintained in RPMI 1640 supplemented with 10% FBS.

### 2.10 Antibodies and reagents

DCP2, abs118391, Absin, 1:1000(WB), 1:75 (IHC); NUDT16:12889-1-AP, Proteintech, 1:1000 (WB), 1:50 (IHC), and β-actin from abclonal, 1:4000 (WB).

### 2.11 Tissue microarray

To explore the expressions of DCP2 and NUDT16 in Ovarian cancer tissues and paired migration tissues, tissue microarray containing 48 OC tissues and 48 migration tissues were obtained from the surgical operations of A Department of Gynecology Oncology, National Cancer Center/National Clinical Research Center for Cancer/Cancer Hospital. All included patients received operation between January 2010 and October 2019. Eliminating 11 ineffective OC tissues, a total of 81 OC cases included in these two studies.

### 2.12 CCK8 (cell proliferation and cell activity detection)

In this study, CCK8 experiments were carried out in 96-well plates. Before each test, CCK8 and culture medium were mixed at 1:10,100 and were added to each well with 50μ. After incubation at 37°C for one hour, the absorbance of each hole at 450 nm was measured. When detecting cell proliferation, the number of inoculated cells is 1.5 per well×10^3^ cells, a total of 5 plates, were tested at the same time every day for 5 consecutive days, and finally the cell proliferation curve was drawn. When testing cell activity, the number of inoculated cells is 1 per well×10^4^ cells were treated according to the experimental requirements, and then tested after 24 hours to calculate the changes in cell activity.

### 2.13 Plate cloning experiment

In this study, the plate cloning experiment was completed in a six-well plate, and the number of A2780 and 3AO cells was 1.5 per well×103 cells were incubated in 37°C incubator for 9**–**12 days after inoculation, until visible monoclone was formed. When collecting, wash with PBS, fix with methanol for 15 minutes, discard and dye with crystal violet.

### 2.14 Western blotting

Western blot was performed according to the standard protocol. Briefly, cells were harvested and lysed in RIPA buffer (1% NP-40, 0.1% sodium dodecyl sulfate (SDS), 50 mM Tris–HCl pH 7.4, 150 mM NaCl, 0.5% sodium deoxycholate, 1 mM (EDTA), 1×proteinase inhibitor cocktail (Roche)) for 30 min on ice. The proteins were resolved on 10% SDS-PAGE and transferred onto PVDF membranes (Millipore). The membranes were blocked with 5% milk powder solution, then incubated with specific antibodies at 4**°**C overnight. Following incubation with secondary antibodies, immunoblots were visualized using the ImageQuant LAS-4000 System (GE). Antibodies for western blotting are listed in ‘Antibodies and reagents’.

### 2.15 Immunohistochemistry assay

Tissue microarrays were stained with anti-DCP2(abs118391, Absin) and anti-NUDT16(12889-1-AP, Protientech) antibodies. The representative images of IHC staining were captured by Aperio ScanScope (Leica, Nussloch, Germany).

### 2.16 Statistics

Statistical analysis was done in the R software (version 3.5.2). Correlations between sample groups and clinical variables were assessed using the Student’s t-test for continuous variables. Significance was set as *p<0.05, **p<0.01, ***p<0.001.

## 3 Results

### 3.1 Consensus clustering of m7G genes in two clusters with different immune pattern of OV

**Figure 1:**
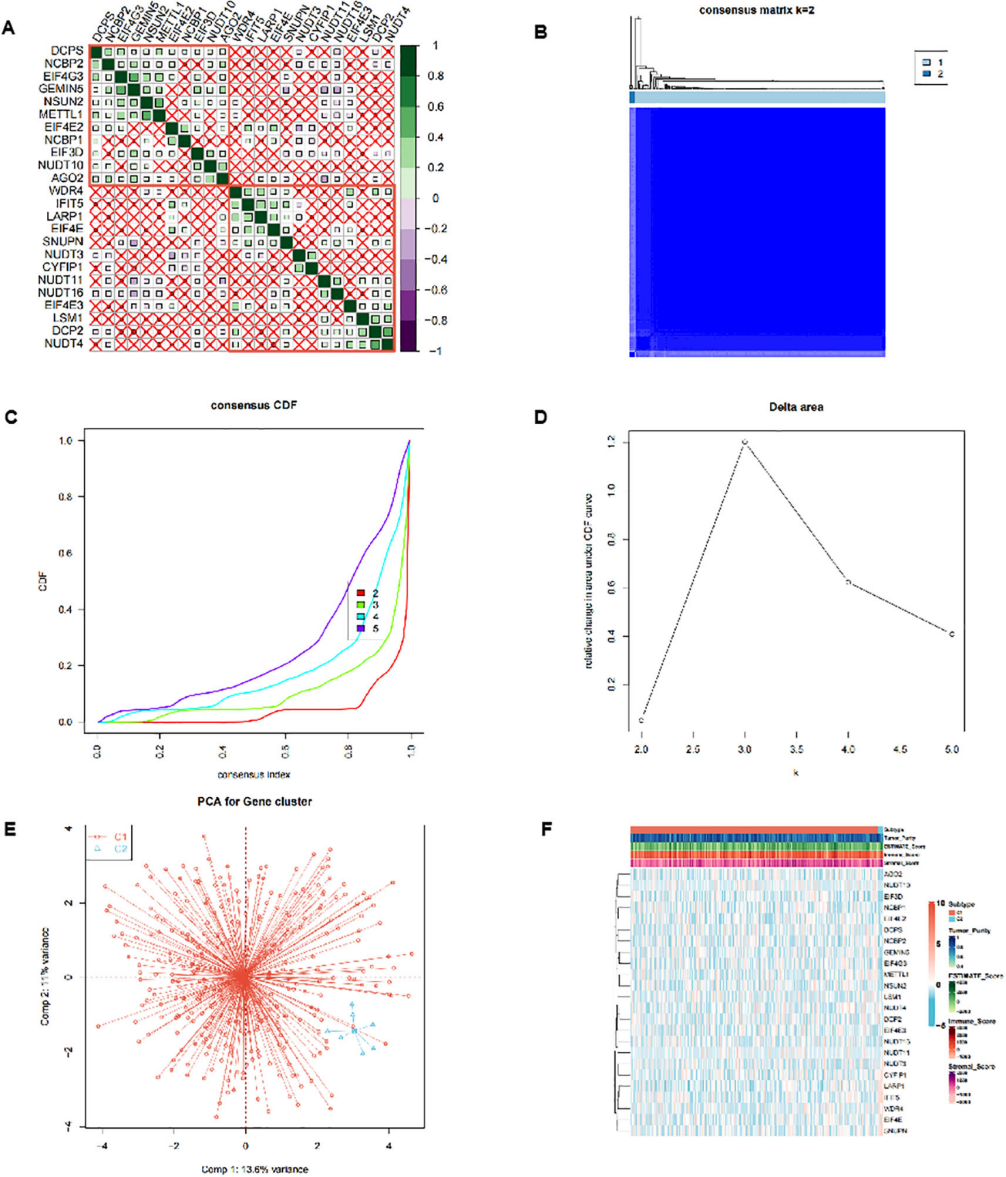
Cluster analysis of m7G-related genes. **(A)** Correlation analysis between the 24 m7G-related genes in the TCGA-OV dataset. **(B-D)** Consensus cluster analysis divided 352 samples into 2 subtypes. **(E)** Principal component analysis (PCA) of 2 subtypes. **(F)** Heatmap of expression profiles of 24 genes between 2 subtypes.

**Figure 2:**
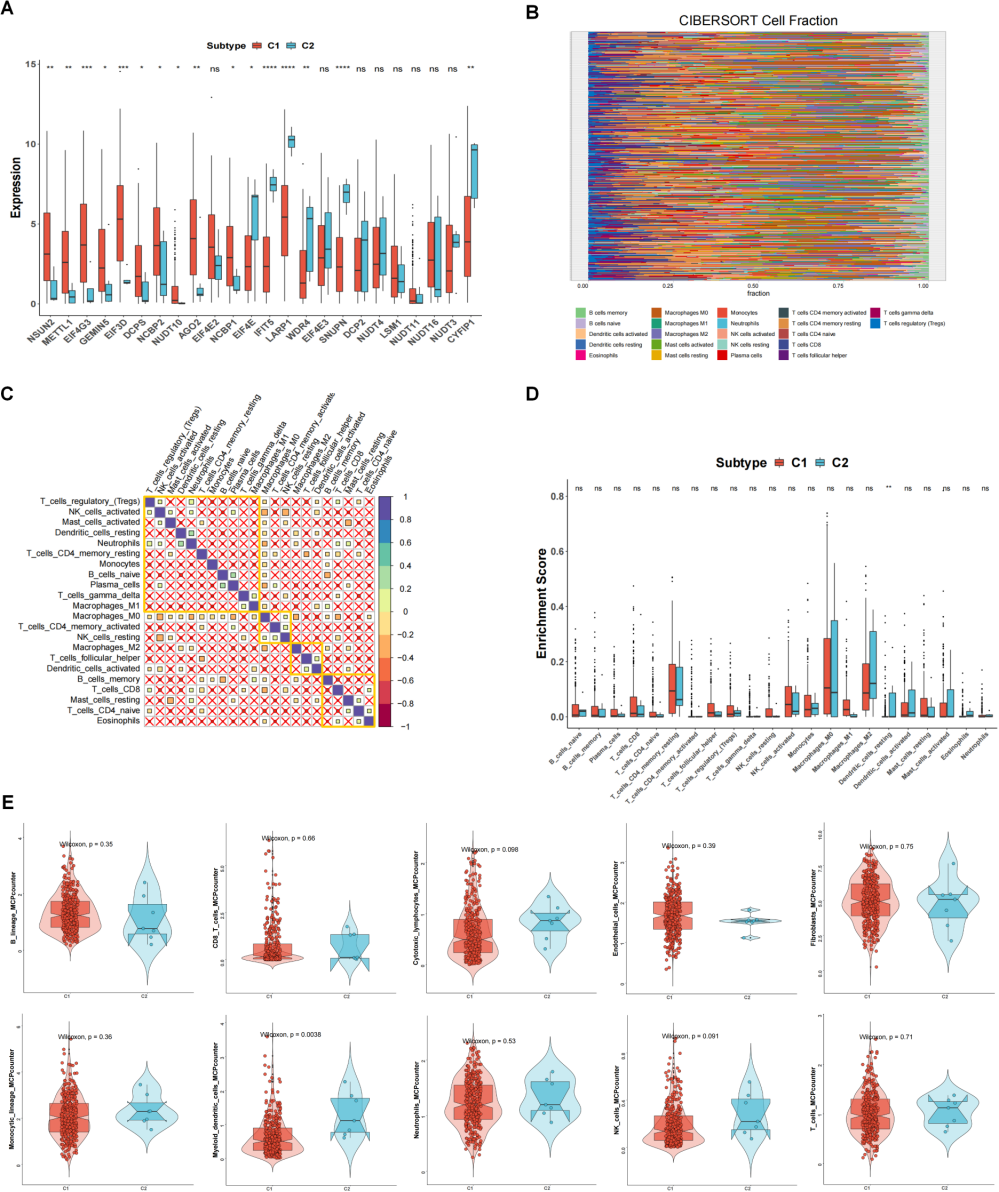
The relationship between 2 clusters and immune infiltrations. **(A)** The expression differences of 24 m7G-related genes in different subtypes. **(B)** Degree of 22 immune cells infiltration in different samples in CIBERSORT results. **(C)** Correlation of 22 immune cells. **(D)** Content of immune cells in different subtypes. **(E)** MCPcounter results of immune cells between different subtypes. *p<0.05, **p<0.01, ***p<0.001; ns means no significance.

In the TCGA-OV dataset, the correlation analysis of 24 m7G-related genes showed that there was a close correlation between these genes (**Figure 1A**) (p<0.05). Consensus cluster analysis divided the 352 samples into 2 subtypes (**Figure 1B**). CDF curve of **Figure 1C** shows the cumulative distribution function when K takes different values. It is used to judge the value of K, and CDF reaches the approximate maximum value. During this time, the cluster analysis results are the most reliable, usually taking the value of K with a small decline slope of CDF. Relative change in area under CDF curve was exhibited in **Figure 1D**. The principal component analysis (PCA) showed that the 2 subtypes could be clearly distinguished (**Figure 1E**), indicating there are significant differences in the expression profiles between different subtypes. At the same time, the expression values of 24 genes were significantly different between the two subtypes, indicating that our typing results have good stability and accuracy (**Figure 2A**). The results of CIBERSORT showed that the infiltration degree of 22 types of immune cells was different between different samples (**Figure 2B**), and there was a significant correlation between Macrophages_M0 and most other immune cells in the 22 types of immune cells (**Figure 2C**), indicating that these cells were in the synergy of OV patients. and the content of most immune cells differed significantly among the different subtypes (**Figure 2D**). At the same time, the results of MCPcounter showed (**Figure 2E**) that Myeloid dendritic cells were significantly different among different subtypes (p <0.05), indicating that Myeloid dendritic cells between subtypes had significantly different contents.

### 3.2 The interaction and correlation among the m7G regulators in two patterns

**Figure 3:**
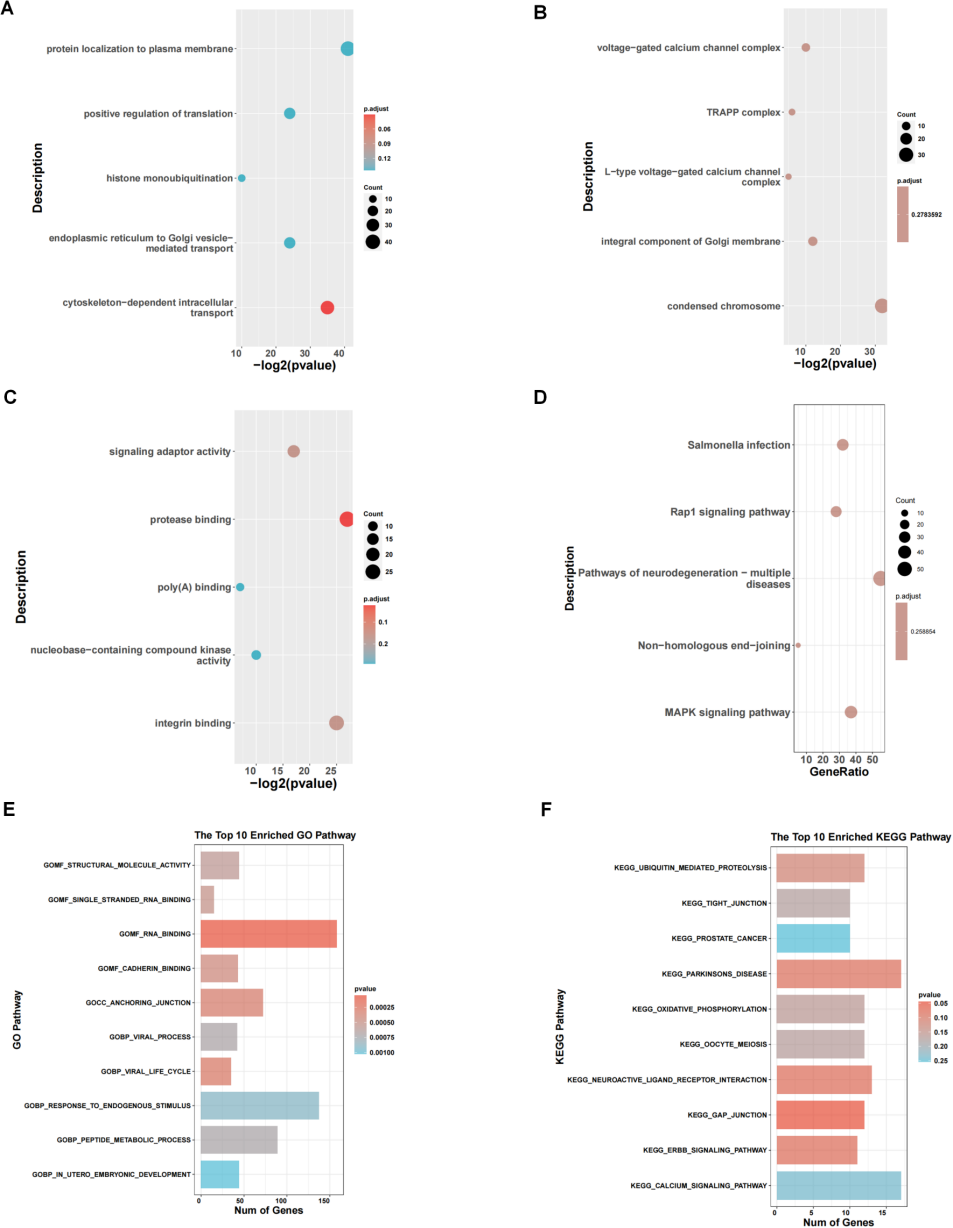
Pathway enrichment analysis of 2 clusters. **(A-C)** GO enrichment analysis between subtype 1 and subtype 2. **(D)** KEGG enrichment analysis. **(E, F)** Results of GSEA concerning GO and KEGG pathways.

We performed enrichment analysis between subtype 1 and subtype 2, and the GO enrichment analysis results showed that the differential genes between subtypes were mainly enriched in different biological process (BP), cellular component (CC), and molecular function (MF). In **Figure 3A**, they showed enrichment in cytoskeleton-dependent intracellular transport (red circle, p <0.05). In **Figure 3B**, there is no significant difference in CC. In **Figure 3C**, they were enriched in protease binding. KEGG enrichment analysis results (**Figure 3D**) showed that the differential genes between subtypes were mainly enriched in MAPK signaling pathway, non-homologous end-joining and Rap1 signaling pathway. GSEA results (**Figures 3E, F**) showed that the differential genes between subtypes were mainly enriched in GOMF_STRUCTURAL_MOLECULE_ACTIVITY, EGG_TIGHT_JUNCTION and other pathways.

### 3.3 Prognostic analysis of risk model and m7G genes

**Figure 4:**
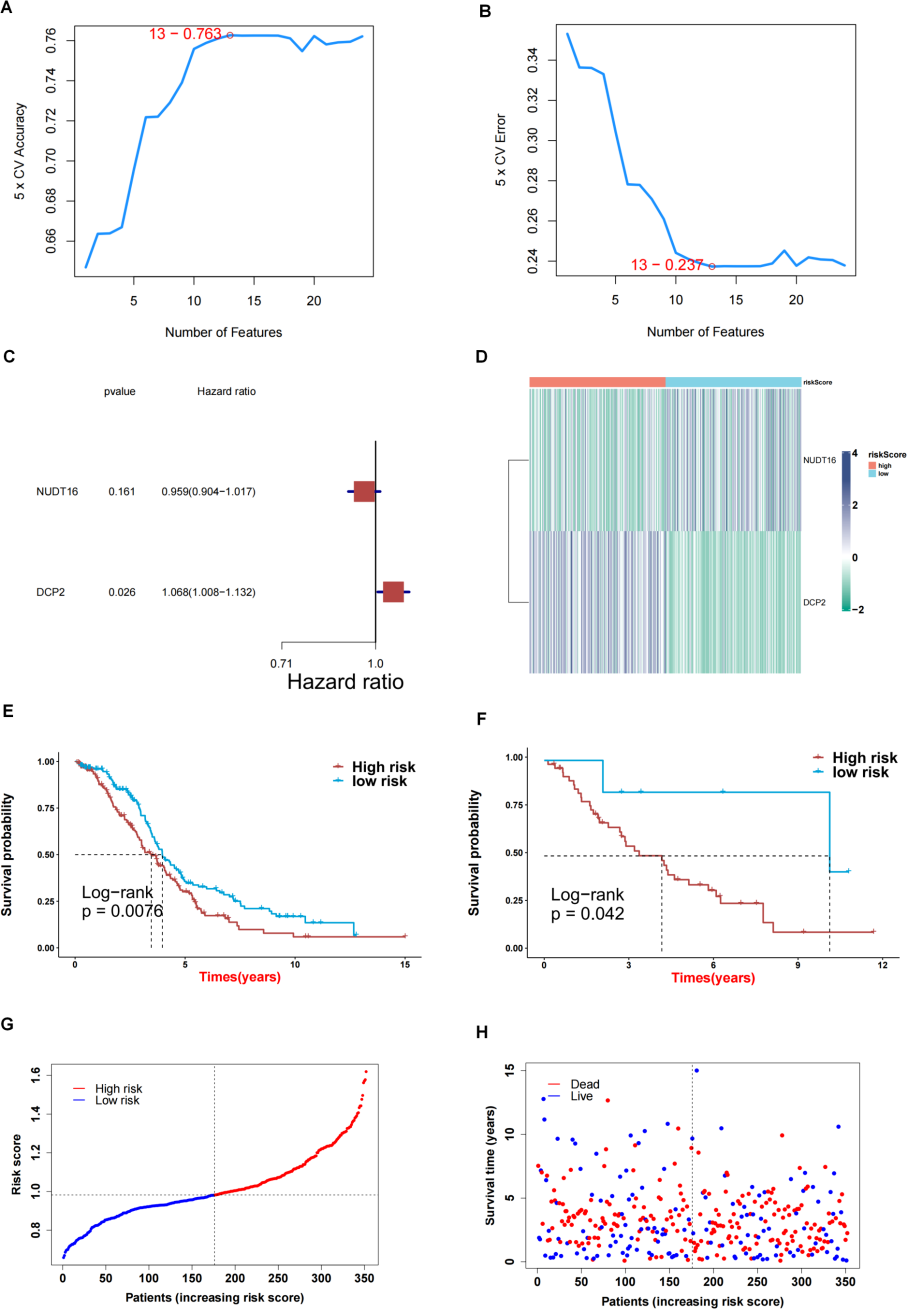
Screening key genes and construction of risk score model by 2 key genes. **(A, B)** Support vector machine algorithm obtained 13 genes associated with prognosis. **(C)** 2 genes for construction of prediction model. **(D)** Heatmap of expression profiles of 2 genes for construction of prediction model in high and low risk groups. **(E)** Survival difference between high and low risk group samples in TCGA-OV. **(F)** Survival difference between high and low risk group samples in GSE30161 database. **(G, H)** Evaluation of the accuracy of risk score.

**Figure 5:**
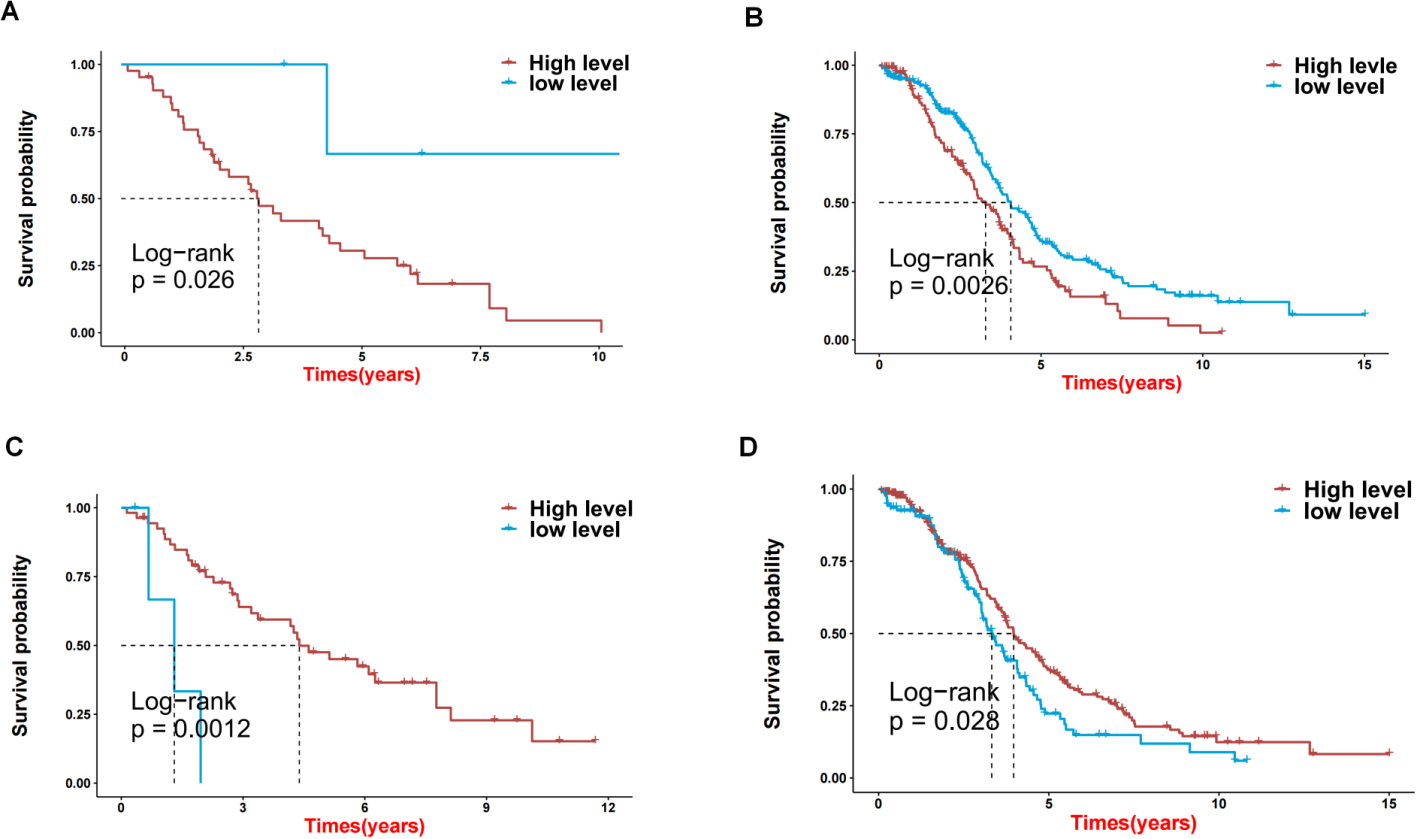
The effects on prognosis of 2 key genes. **(A-D)** Survival analysis based on high and low expression of DCP2 and NUDT16 in GSE30161 **(A, C)** and TCGA-OV **(B, D)**.

First, we obtained 13 genes associated with prognosis through the support vector machine algorithm (**Figures 4A, B**), and then, based on these 13 genes, we performed multivariate cox regression analysis, and identified 2 genes for constructing the prediction model (**Figure 4C**) to get the risk score of each sample in TCGA-OV. Finally, according to the median of the risk score, the samples are divided into high and low risk groups. The survival analysis shows that the survival difference between the high and low risk groups is significant (**Figure 4E**). The difference in survival between high- and low-risk group samples in the validation set GSE30161 was also significant (**Figure 4F**). **Figure 4G** indicated the accuracy of risk score in differentiate high-risk and low-risk patients. **Figure 4H** showed that dead samples had a shorter survival time compared to live samples. At the same time, the expression values ​​of the two genes that constructed the prediction model were also significantly different in the high- and low-risk groups (**Figure 5D**). Single-gene survival analysis was performed in GSE30161 (**Figures 5A, C**) (genes DCP2 and NUDT16) and TCGA-OV (**Figures 5B, D**) (genes DCP2 and NUDT16) based on the expression levels of the two genes, respectively. The results showed that, the single-gene survival analysis of the two genes was significantly different between the high and low expression groups, further indicating that these two genes were significantly related to the prognosis of OV.

### 3.4 Assessment of two m7G-related genes as independent OV prognostic factors

**Figure 6:**
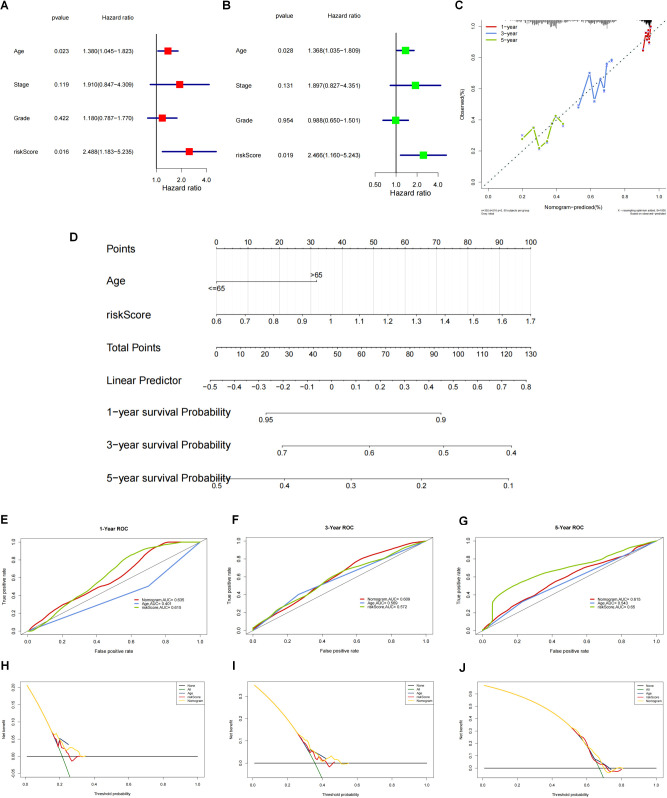
Construction and validation of nomogram based on the risk score and clinical characteristics. **(A)** One-way cox regression prognostic analysis of the scores with other clinical characteristics in TCGA-OV. **(B)** Multi-factor cox regression analysis. **(C)** The accuracy of nomograms to predict prognosis was validated by calibration curves and ROC curves. **(D)** Construction of nomograms based on these 2 clinical characteristics (age and risk score) to predict patient survival at 1, 3 and 5 years. **(E-G)** The ROC curve results showed that the ROC curve AUC values were higher at 1 year **(E)**, 3 years **(F)** and 5 years **(G)**. **(H-J)** For the column line graph and the decision curve analysis at 1 year **(H)**, 3 years **(I)** and 5 years **(J)**. The column line graph showed that the column line graph could predict the prognosis of patients very well.

To perform an in-depth analysis of the prognostic value of the risk score, we performed a prognostic analysis of scores and other clinical characteristics in the TCGA-OV cohort, and univariate cox regression analysis showed (**Figure 6A**) that both age and risk score were significantly associated with prognosis (p<0.05). Further multivariate cox regression analysis confirmed (**Figure 6B**) that age and risk score were still significantly associated with prognosis (p<0.05). Therefore, we constructed a nomogram (**Figure 6D**) based on these 2 clinical features to predict the 1-, 3-, and 5-year survival rates of patients, and verified the accuracy of the nomogram in predicting prognosis through calibration curves and ROC curves. The calibration curve results show (**Figure 6C**) that the predicted values of 1 year, 3 years and 5 years have little deviation from the diagonal line in the figure. The ROC curve results show that in the nomogram’s 1 year (**Figure 6F**) and 5-year (**Figure 6G**) ROC curve, AUC values were higher, and the 1-year (**Figure 6H**), 3-year (**Figure 6I**) and 5-year (**Figure 6J**) decision curve analysis of the nomogram showed that the nomogram can predict the prognosis of patients very well. The above three test methods all show that the nomogram has good accuracy in predicting the prognosis of patients.

### 3.5 Network analysis and immunotherapy response prediction

**Figure 7:**
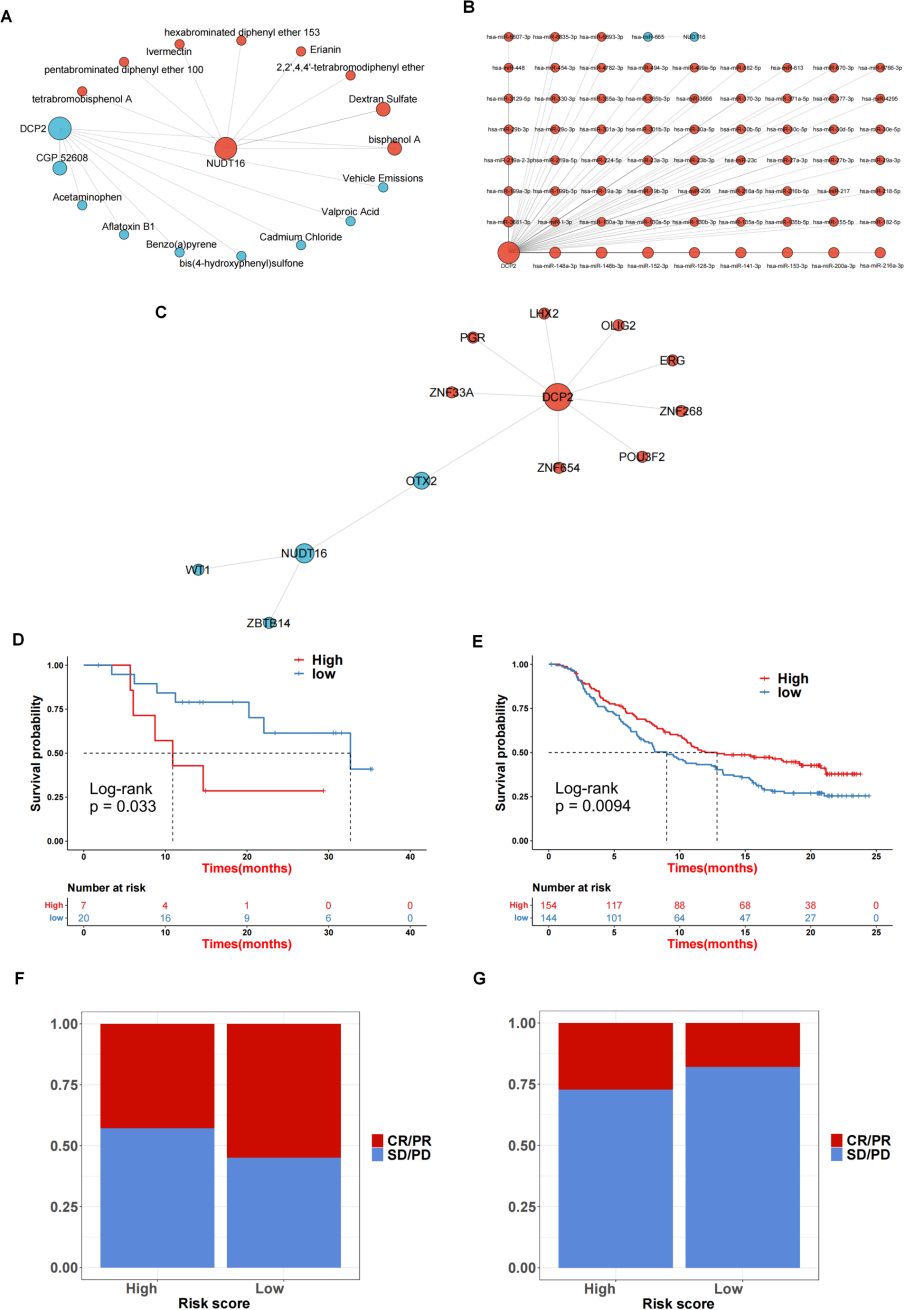
Network analysis and immunotherapy response prediction. **(A)** Compound network of the genes. **(B)** The miRNA network for 2 genes. **(C)** A transcription factor network of the genes was constructed. **(D, E)** The survival rate in GSE78220 **(D)** and IMvigor210 **(E)**. **(F, G)** The correlation between sample survival rate and response to immunotherapy.

We performed network analysis for the two genes for which the predictive model was constructed. First, a compound network of genes was constructed (**Figure 7A**). Here, 8 targeting compounds were predicted for the DCP2 gene, and 8 targeting compounds were predicted for the NUDT16 gene. Then the miRNA network of the genes was constructed (**Figure 7B**), 77 targeted miRNAs were predicted for DCP2 gene, and 1 targeted miRNA was predicted for NUDT16 gene. Finally, the transcription factor network of the gene was constructed (**Figure 7C**). Nine targeted transcription factors were predicted for DCP2 gene, and four targeted transcription factors were predicted for NUDT16 gene. The immunotherapy response of the high and low score groups was also further investigated based on the GSE78220 and IMvigor210 datasets, and we found that the survival of the high and low score groups was significantly different in GSE78220 (**Figure 7D**) and IMvigor210 (**Figure 7E**). There was a correlation between immunotherapy responses and survivals (**Figures 7F, G**), i.e., the higher the survival of the sample, the worse its response to immunotherapy.

### 3.6 Functional verification of 2 key genes and external verification of their impact on prognosis

**Figure 8:**
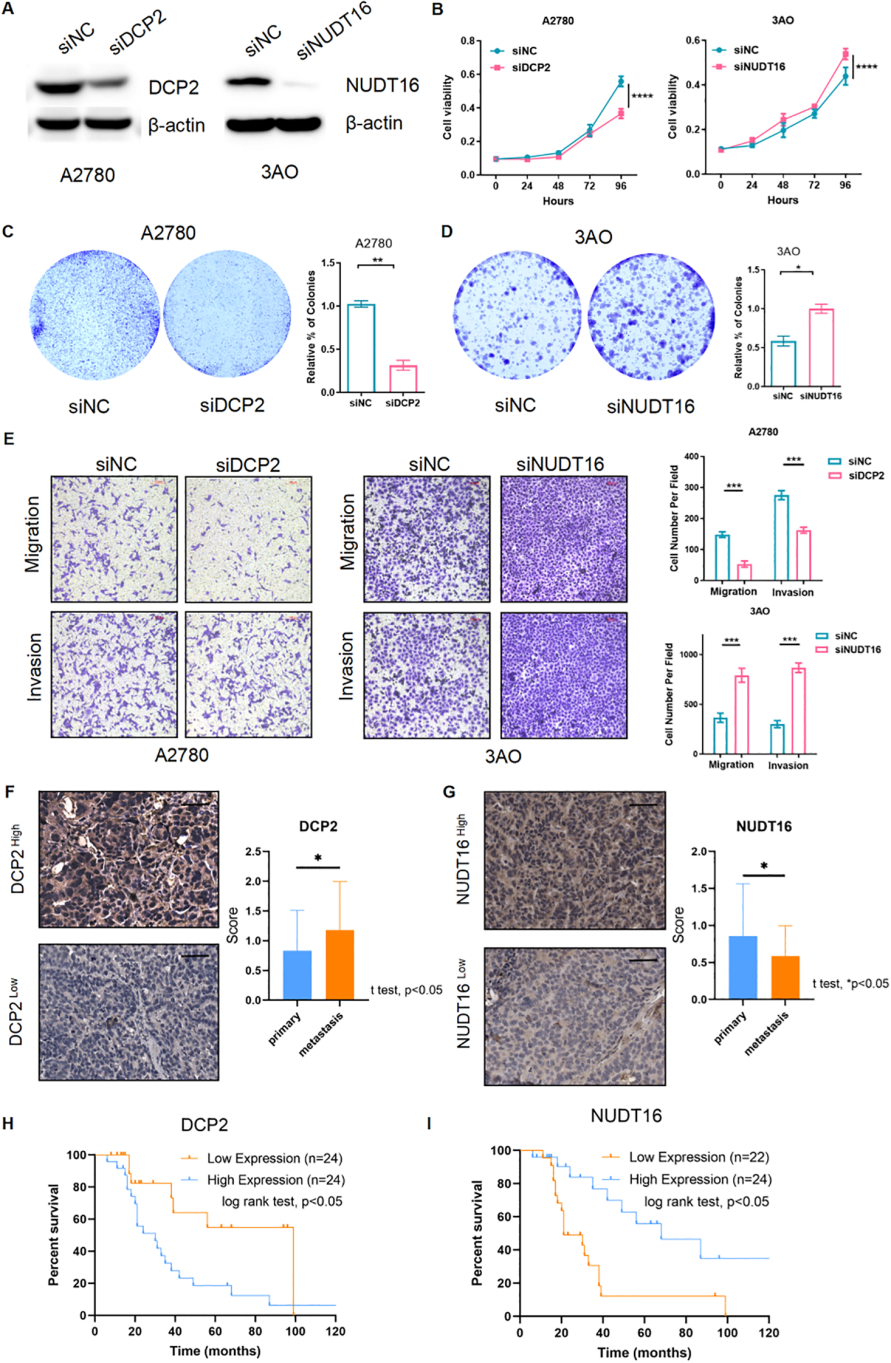
Functional verification of 2 key genes and external verification of their impact on prognosis. **(A)** Western blotting detected the siRNA’s efficiency of the two m7G genes (DCP2 and NUDT16) at the protein level. **(B)** CCK8 method was used to detect the cell proliferation after siRNA knockdown of DCP2 and NUDT16. **(C, D)** The plate cloning method directly observed the formation of cell clones. **(E)** Results of the transwell experiment. **(F, G)** The high and low expression of the two genes in the tissue microarray immunohistochemistry of ovarian cancer patients were performed respectively, Scale bar, 50μm, and the expression of the two genes in the primary and metastatic foci were analyzed. **(H, I)** Two genes in tissue microarray data were analyzed for survival. ****p < 0.0001; ***p < 0.001; **p < 0.01; *p < 0.05.

**Figure 9:**
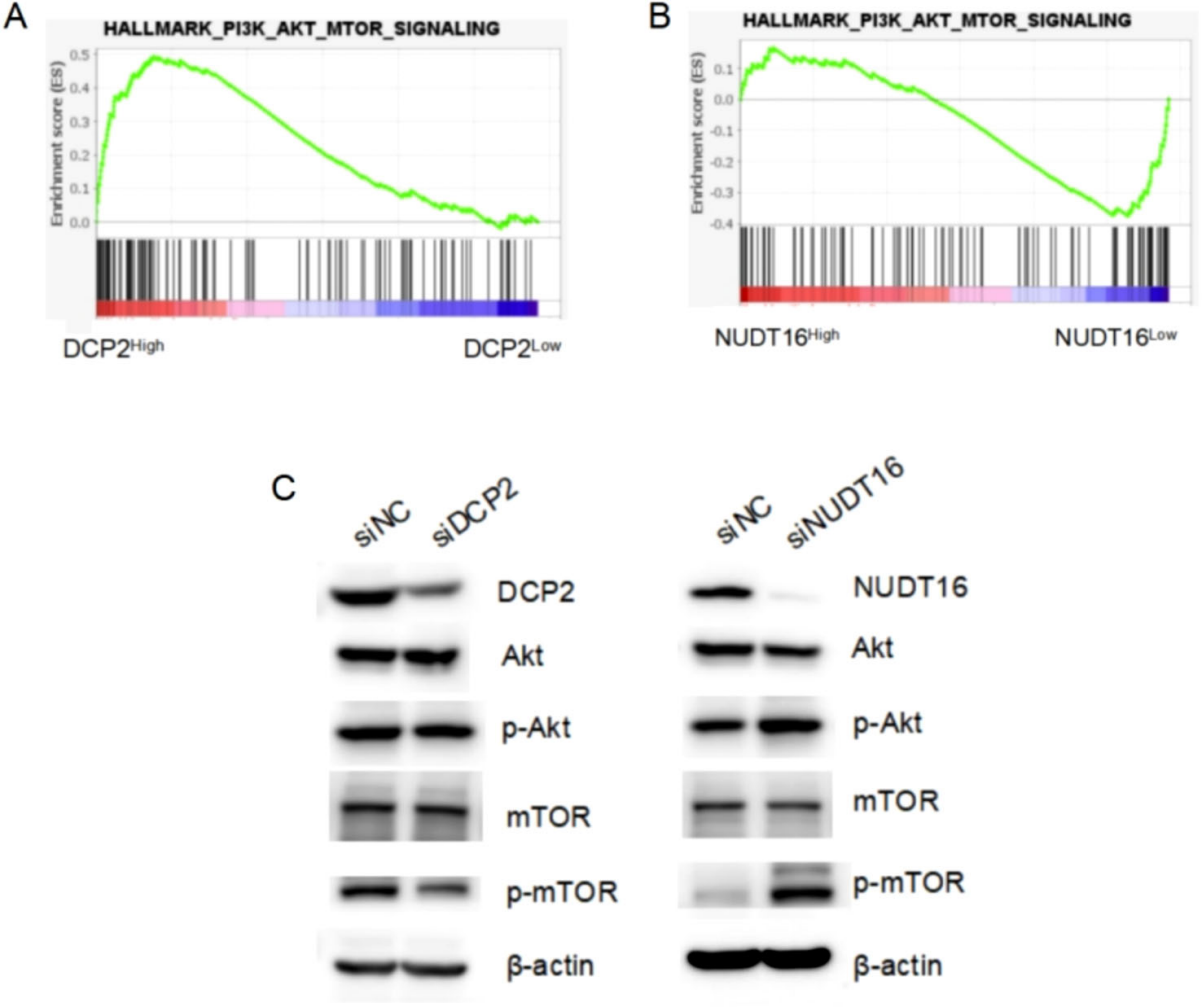
GSEA analysis results and Western Blotting experimental verification. **(A, B)** The activated PI3K-Akt-mTOR signaling pathway in OV patient samples with higher DCP2 expression and lower NUDT16 expression through TCGA database. **(C)** Western blotting detected the expression of key proteins of the PI3K-Akt-mTOR signaling pathway at the protein level, after siRNA knockdown of DCP2 and NUDT16.

To verify the authenticity of the above bioinformatics analysis, we performed some experimental validation. Two key genes were knocked down using siRNA: DCP2 and NUDT16, and the protein-level knockdown efficiency was determined by western blotting experiments (**Figure 8A**). Furthermore, we confirmed that PI3K-Akt-mTOR signaling was activated in OV patient samples with higher DCP2 expression and lower NUDT16 expression and western blotting were performed to verify the expression of key proteins of the pathway when knocking down two genes, and the results were basically consistent with what we expected (**Figure 9**). The proliferation of cells after SI knockdown of DCP2 and NUDT16 at 0-24-48-72–96 hours was detected using CCK8 method and plotted as a line graph, which showed that the cell proliferation was reduced after knockdown of DCP2 and enhanced after knockdown of NUDT16 (**Figure 8B**). The flat panel cloning method visually shows that the number of clonogenic cells from ovarian cancer cells knocked down by DCP2 is smaller than that of the control group, while the clonogenic ability of cells knocked down by NUDT16 is diminished (**Figures 8C, D**). We performed relevant transwell assays to verify the migratory invasion ability of these two genes after knockdown, and the migratory invasion ability became poor after knockdown of DCP2, while the migratory invasion ability was improved after knockdown of NUDT16 (**Figure 8E**). Two tissue microarrays from surgical patients with good clinical prognostic information were immunohistochemically stained for two key genes, respectively, showing high and low gene expression. We scored the primary and metastatic foci separately in tissue microarrays and analyzed the relationship between these two genes and metastases at the clinical sample level with positive statistical results (**Figures 8F, G**).The k-m curves obtained from the analysis of immunohistochemical staining of tissue microarrays and clinical prognostic information showed that high expression of DCP2 was associated with poor prognosis, while high expression of NUDT16 improved the survival of ovarian cancer patients(**Figures 8H, I**).

## 4 Discussion

Ovarian cancer is the most lethal gynecological malignancy in the world. Existing therapies cannot significantly improve the long-term prognosis of ovarian cancer. Sometimes, the number of negative clinical trials of platinum-resistant EOC may be frustrating (30–35). Immunotherapy may represent an important therapeutic approach in platinum resistant Ovarian cancer (36). Hence, the validation of predictive to identify the subset of patients who may benefit from immune modulation is an important translational endpoint in ongoing trials. Immune checkpoint therapy represented by PD-1/PD-L1 has achieved significant efficacy in tumors such as melanoma and non-small cell lung cancer, but its response rate in ovarian cancer clinical trials is less than 10% (37, 38), suggesting that the immunosuppressive microenvironment of ovarian cancer is complex. Specific m7G-locus signatures are associated with ovarian cancer (39), so we studied the immune microenvironment of ovarian cancer based on the expression of m7G regulators. Consensus clustering analysis obtained two subtypes, and the immune cell composition between subtypes, including Myeloid dendritic cells, with significantly different contents, suggest that m7G modification may affect the immune microenvironment of ovarian cancer.

In addition to the inflammatory microenvironment, immune escape and immunosuppressive microenvironment are also a major feature of tumors (40). Combination of ESTIMATE, MCPcounter and CIBERSORT algorithms evaluated the immune cell infiltration between different m7G subtypes, covering 22 types of immune cells, as well as immunity scores, stroma scores, and tumor purity, which provided a multifaceted perspective for the analysis of the tumor immune microenvironment. To survive and proliferate in primary and distant organs, tumors must evade immune surveillance and avoid killing by cytotoxic lymphocytes. Tumors do this by remodeling the immune microenvironment into an immune tolerance microenvironment (41). Tumor cells achieve this immune evasion by down-regulating antigen presentation, up-regulating immunosuppressive ligands, and secreting immunosuppressive factors (42). We found that m7G risk scores were also associated with immunotherapy response rates, suggesting that m7g modification may be an effective therapeutic target for improving immunotherapy response rates in ovarian cancer.

Further analysis showed that two m7g regulator genes, DCP2 and NUDT16, could be used to construct a prognostic risk model of ovarian cancer, and that risk scores were significantly associated with ovarian cancer prognosis. The risk score constructed based on DCP2 and NUDT16 is an independent prognostic factor for ovarian cancer, suggesting the important functions of these two genes in ovarian cancer, which undoubtedly deserves further study. The potential for biomarkers DCP2 and NUDT16 in guiding therapy would strengthen the clinical relevance.

DCP2 is a major messenger RNA decarboxylase in eukaryotic cells, and eukaryotic messenger RNAs contain a 5’ cap that functions to protect mRNA from rapid extranuclear degradation. The precise control of the mRNA decapping pathway by DCP2 is very important for the regulation of intracellular mRNA levels (43). Other research shows that miR-4293 regulating WFDC21P through downregulate DCP-2 while miR-4293 promotes tumor cell proliferation and metastasis but suppresses apoptosis (44). However, there is no research reports on the specific function of DCP2 in ovarian cancer. In this study, we found through CCK8 and plate cloning experiments that DCP2 promotes ovarian cancer cell proliferation, and through transwell experiments, we found that DCP2 promotes ovarian cancer cell metastasis. We also found that high expression of DCP2 in ovarian cancer tissue is associated with poor patient prognosis.

Furthermore, we investigated the function of NUDT16 and its association with ovarian cancer prognosis. NUDT16 is a member of the nucleoside diphosphate linker X (Nudix) hydrolase family. Nudix protein is characterized by containing a highly conserved 23 amino acid structure, and NUDT16 is necessary for the stability of some gene expression (45). Studies have shown that NUDT16 plays a certain role in DNA damage-related diseases, such as polyglutamine (polyQ) diseases, including Huntington’s disease (HD) (46). Similarly, we found that NUDT16 exhibited opposite functions from DCP2 in CCK8, plate cloning, and transwell experiments. We also found that high expression of NUDT16 in ovarian cancer tissue is associated with improved patient prognosis. These suggest that high expression of NUDT16 may be a protective factor for ovarian cancer.

Additionally, we explored the mechanisms underlying the research findings. We performed GSEA analysis and found that the PI3K-Akt-mTOR signaling was activated in OV patient samples with higher DCP2 expression and lower NUDT16 expression. In cisplatin-resistant ovarian cancer cells, m7G modification significantly enhances translation of EGFR pathway genes and activates PI3K/AKT/mTOR signaling, which reduces apoptosis sensitivity (47). It was reported that the UBE2S gene could inhibit autophagy by activating the PI3K/AKT/mTOR signaling pathway to induce cisplatin resistance in OV (48). Then, we used Western Blotting to verify the expression of key proteins of the pathway when knocking down DCP2 or NUDT16 (**Supplementary Figure 1**). These results imply that DCP2 and NUDT16 may regulate the malignant progression of ovarian cancer through the PI3K-Akt-mTOR signaling pathway, which may have the potential to develop them as drug targets.

This is the first study to identify DCP2 and NUDT16 as potential biomarkers for in guiding OV therapy. However, there are still some limitations in this study, such as the absence of animal experiments. We will continue to investigate the impact of these two genes on immune cell infiltration in the microenvironment, the correlation between these two genes and m7G, and the lack of in-depth exploration of related mechanisms. In addition, our current data establish correlations but do not establish causality or define the precise signaling nodes through which these genes exert their effects. Future investigations should systematically dissect how these genes modulate the recruitment, activation, or polarization of specific immune cell subsets (e.g., T lymphocytes, macrophages, dendritic cells) within diverse tissue microenvironments. This could involve employing advanced techniques such as single-cell RNA sequencing, immunohistochemistry, and cytokine profiling to map cell-cell communication networks and signaling cascades driven by the genes of interest.

## 5 Conclusion

We discovered for the first time that m7G-related genes play a role in the immune microenvironment of ovarian cancer, which may regulate immune cell infiltration and immune function in ovarian cancer tissue in a synergistic manner. m7G modification affects the cellular composition of the immune microenvironment in ovarian cancer, and two key m7G modification-regulated genes, DCP2 and NUDT16, promotes the proliferation and metastases of ovarian cancer cells, and its high expression is associated with poor prognosis. The prognostic risk model of m7G has excellent predictive performance. And the biological mechanism of their specific functions about m7G in ovarian cancer needs further study. Our work provides new ideas for the mechanism of ovarian cancer and immunotherapy.

## Data Availability

The original contributions presented in the study are included in the article/**Supplementary Material**. Further inquiries can be directed to the corresponding authors.
