# Supplementary figures and images for: Analysis of m7G-related signatures in the tumor immune microenvironment and identification of clinical prognostic regulators in ovarian cancer
Source: Front Immunol. 2025 Aug 14;16:1595618. doi: 10.3389/fimmu.2025.1595618 (PMC12390964; doi:10.3389/fimmu.2025.1595618)

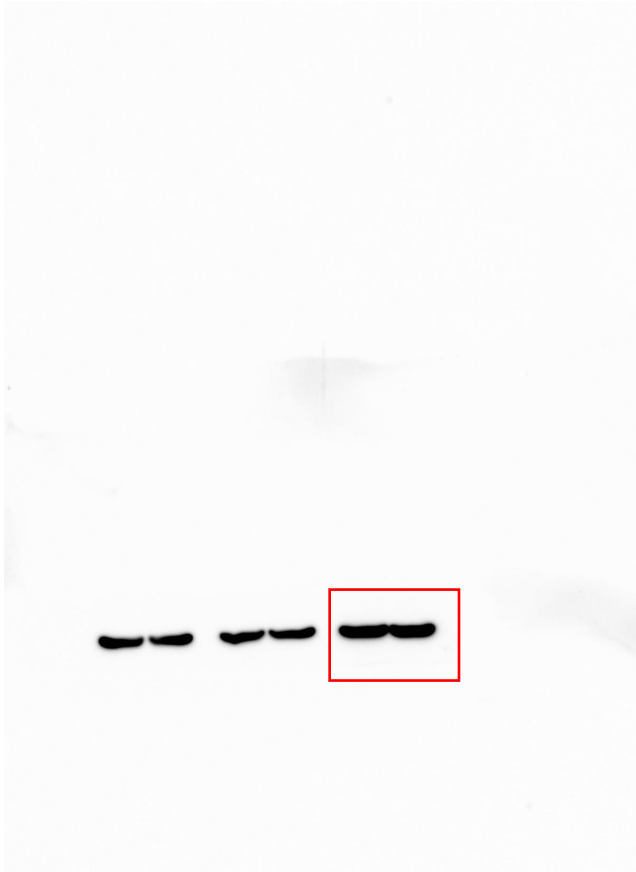

$\beta$ -actin of NUDT16

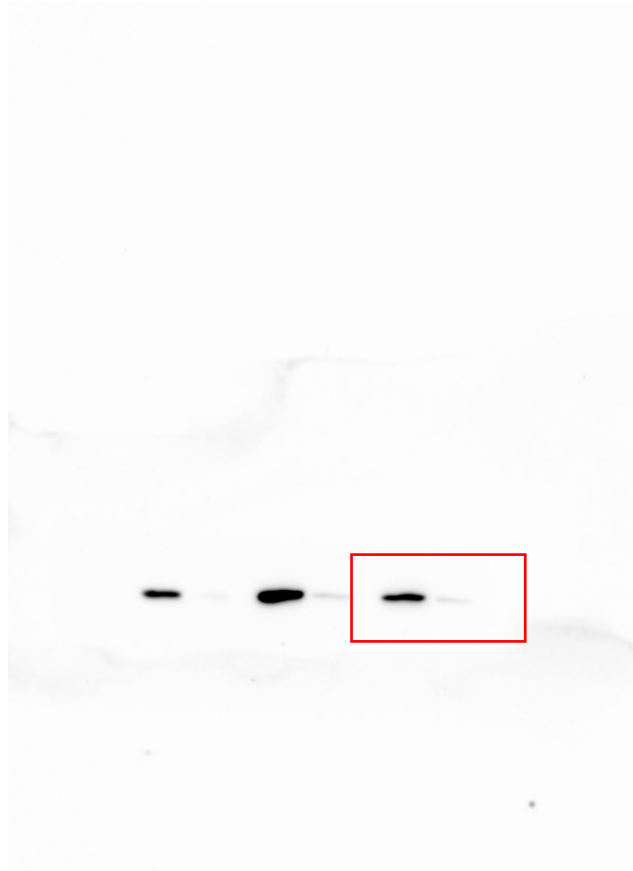

NUDT16

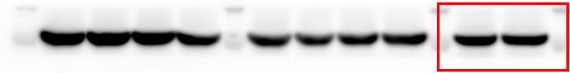

$\beta$ -actin of DCP2

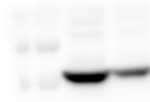

DCP2

Supplement: Supplementary file 1 [file DataSheet1.pdf]

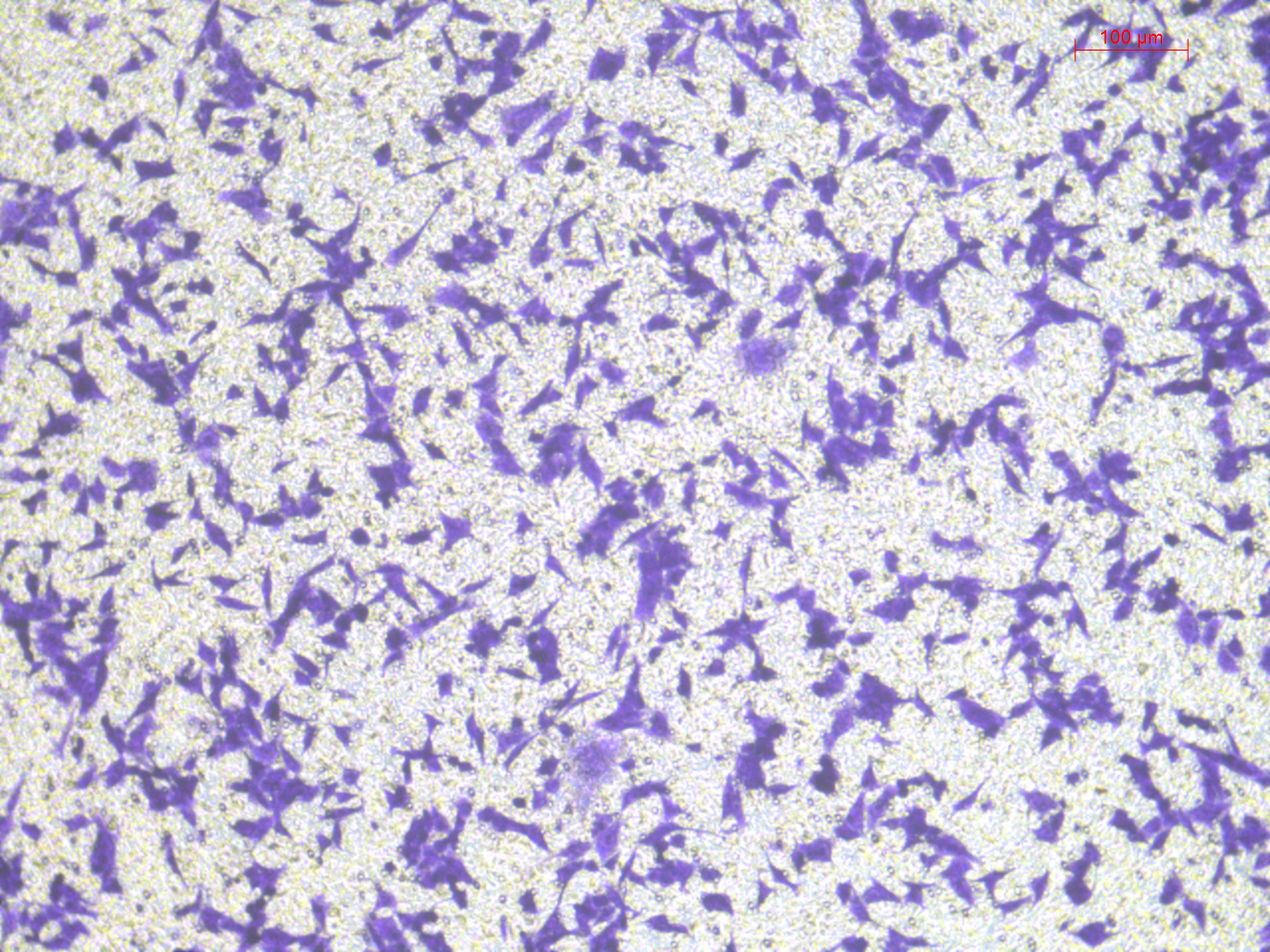

Supplement: Supplementary file 2 [file Image1.tif]

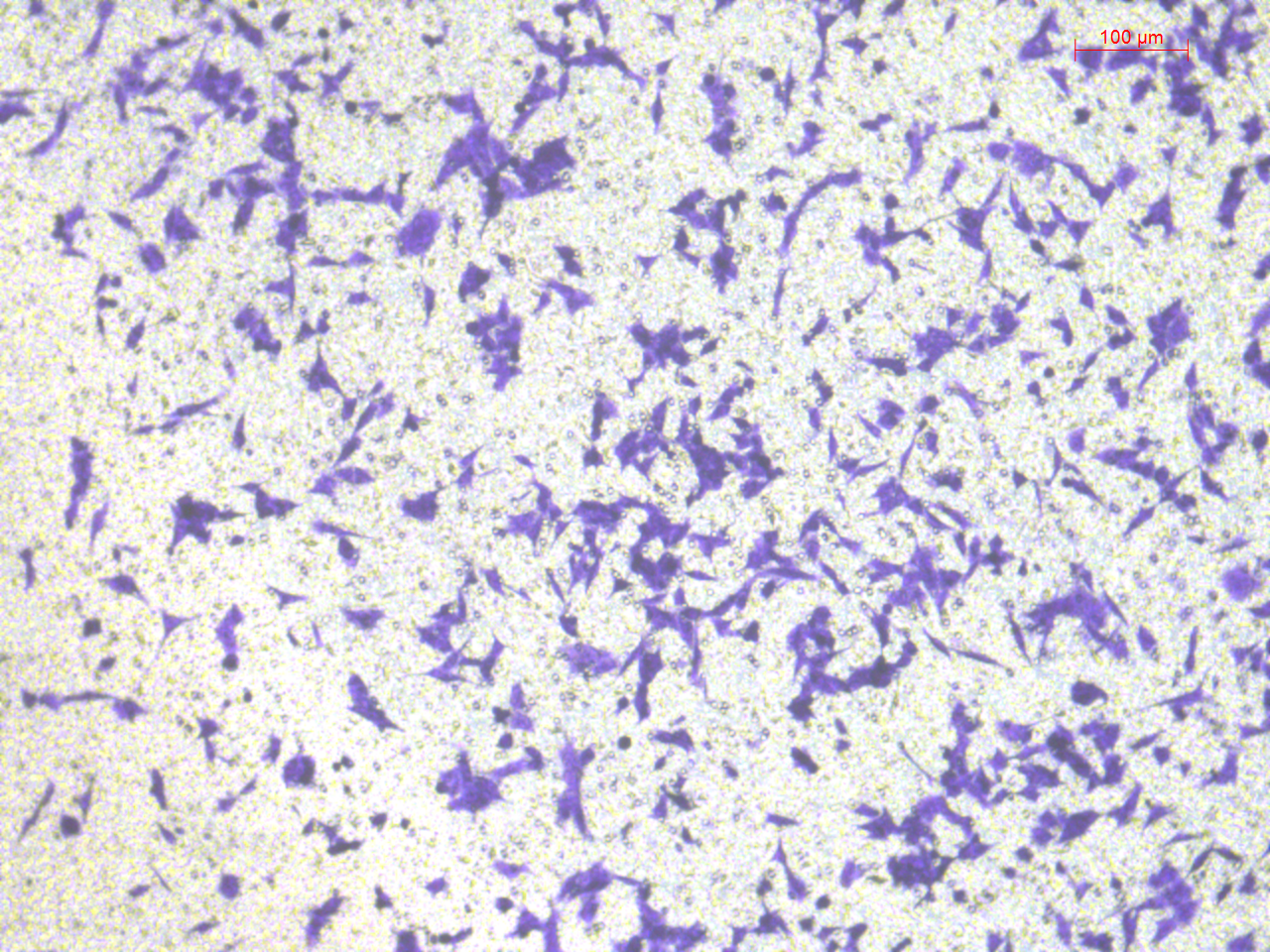

Supplement: Supplementary file 3 [file Image2.tif]

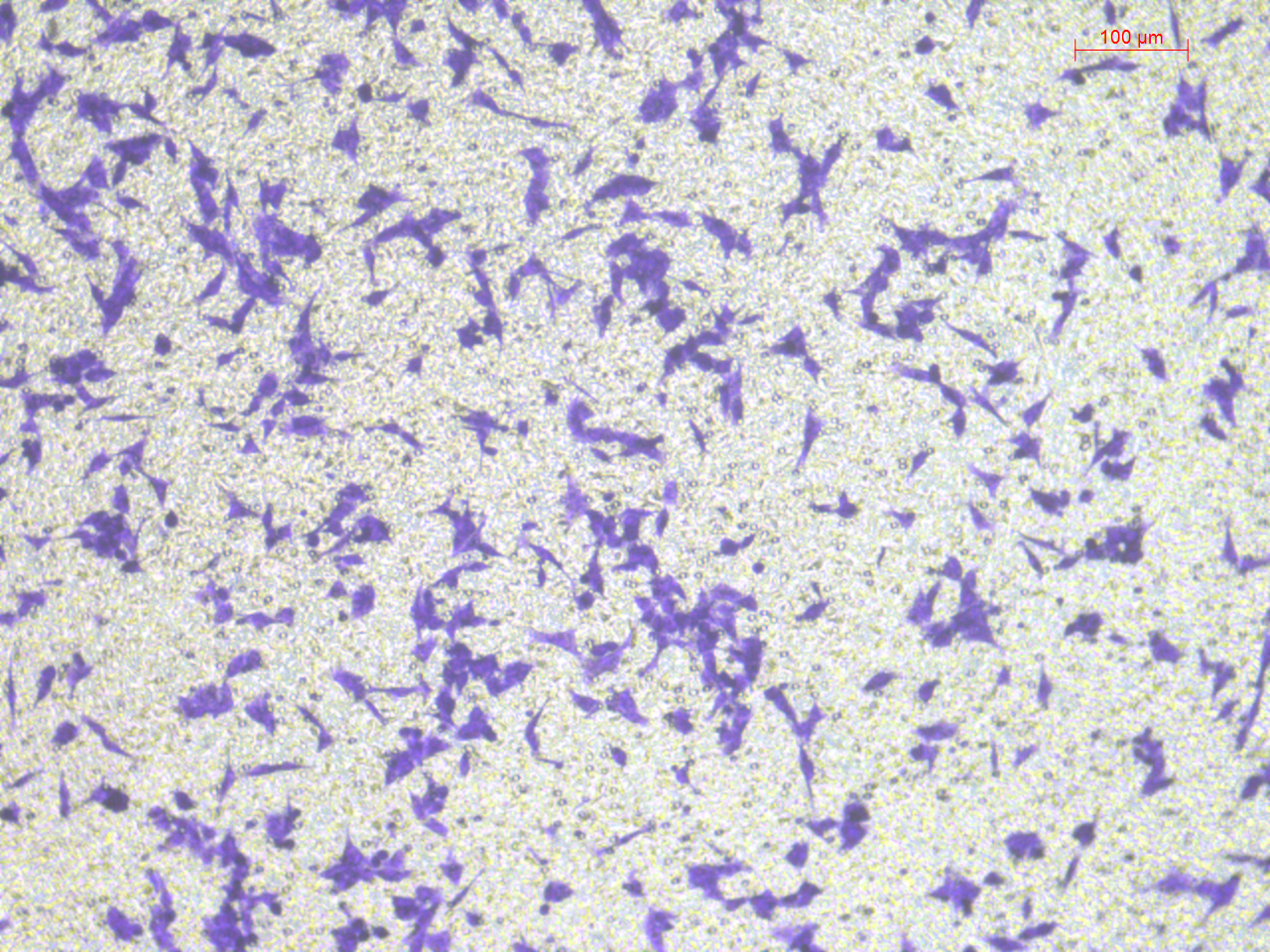

Supplement: Supplementary file 4 [file Image3.tif]

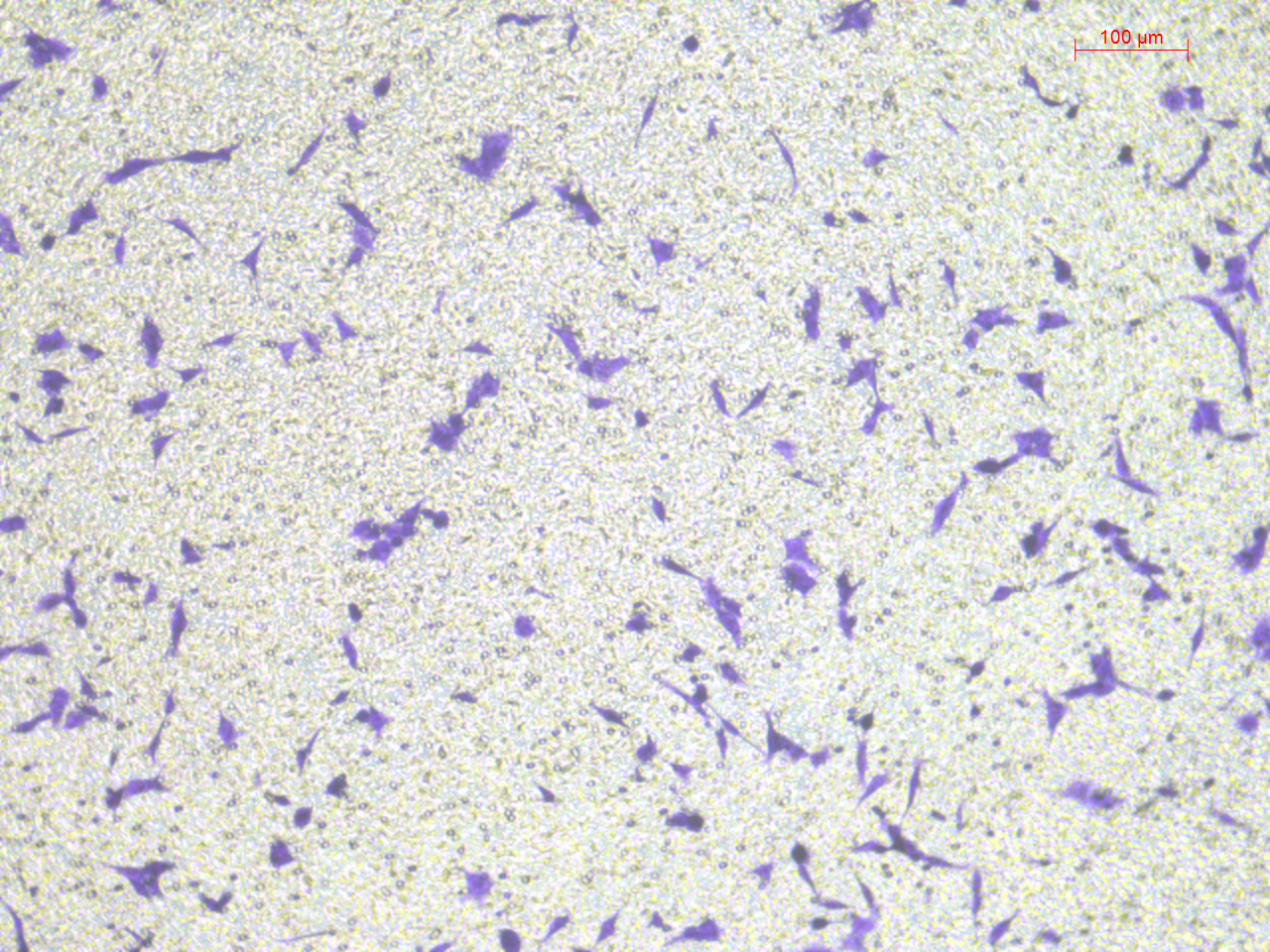

Supplement: Supplementary file 5 [file Image4.tif]

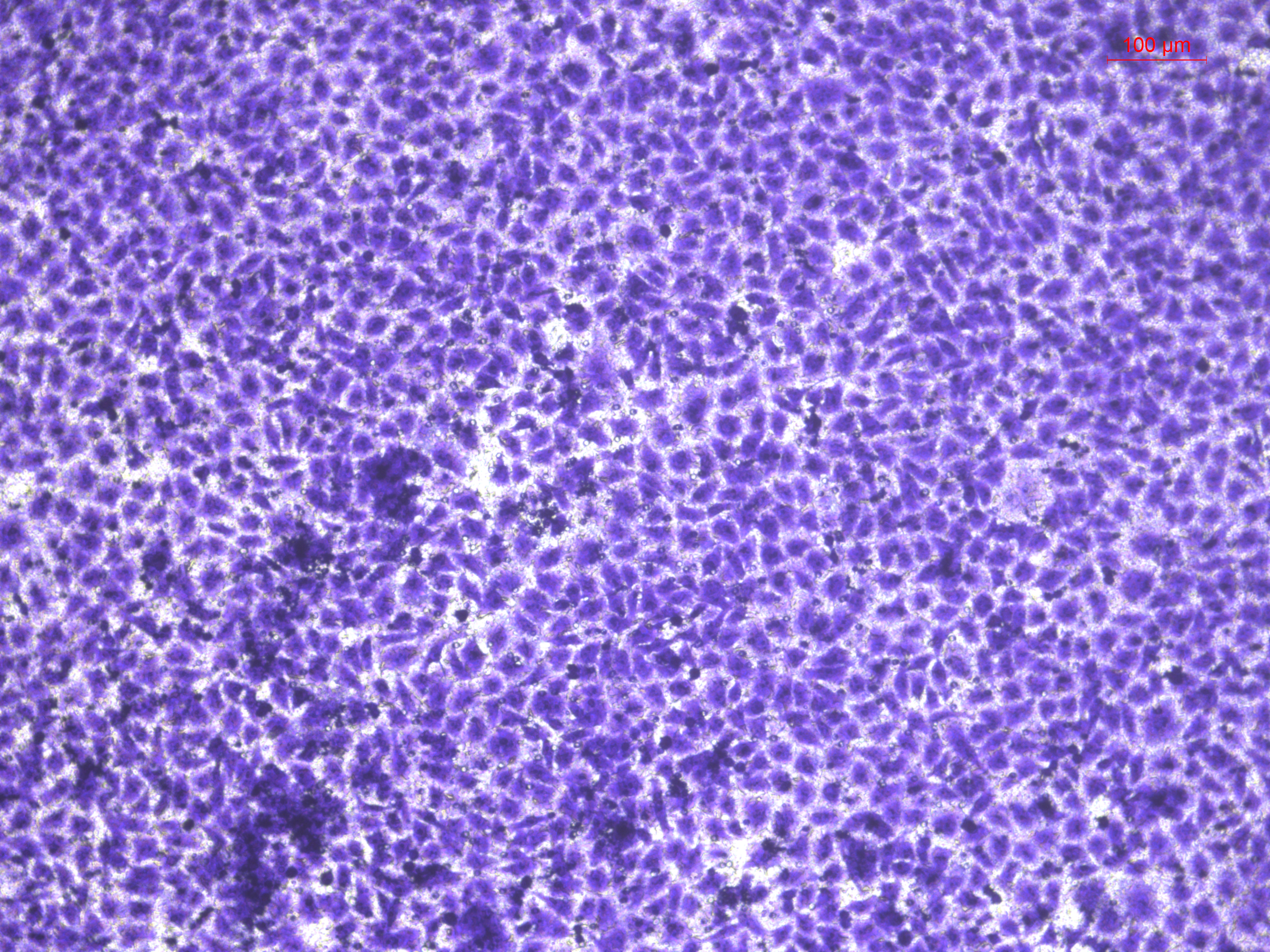

Supplement: Supplementary file 6 [file Image5.tif]

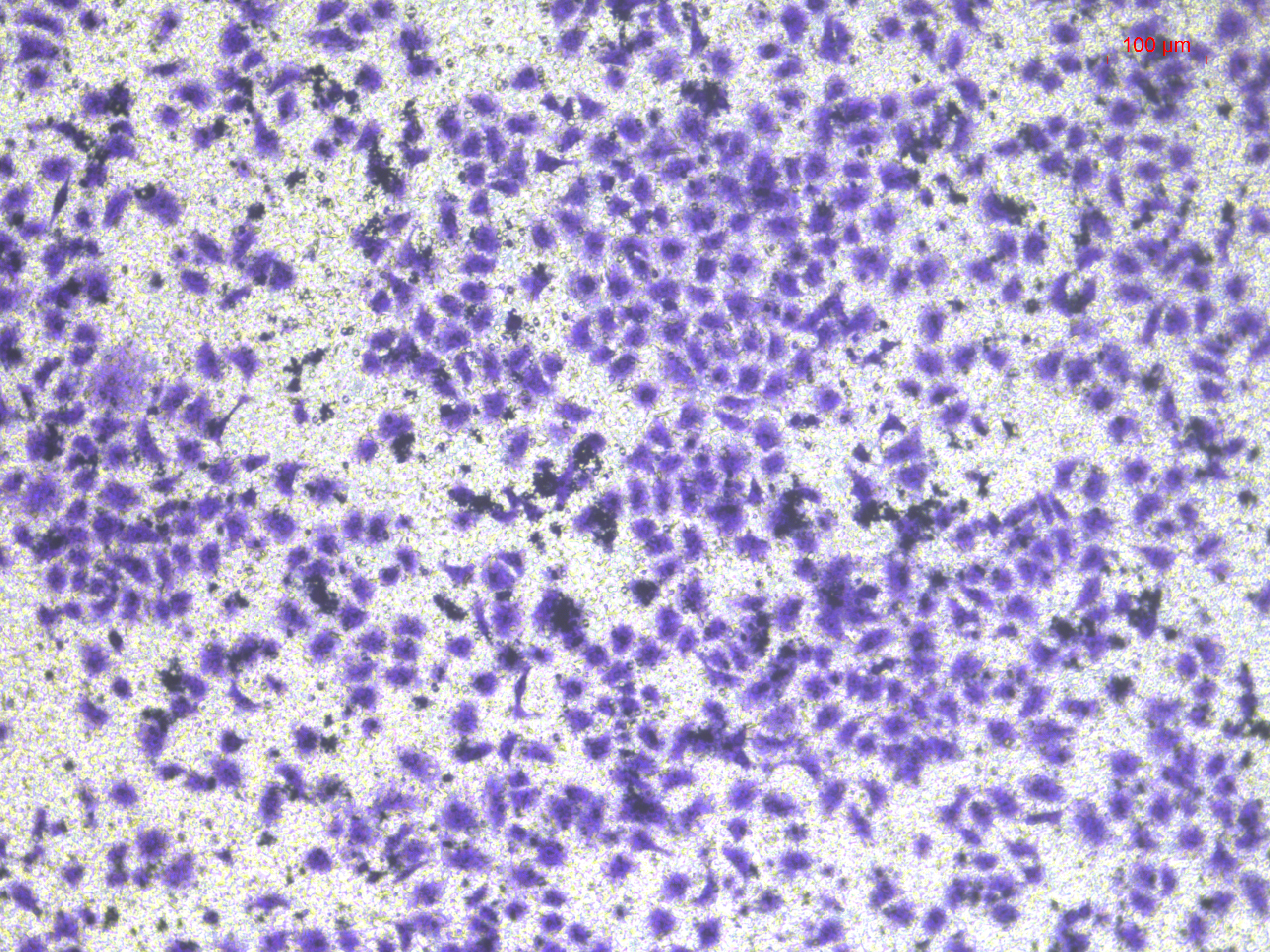

Supplement: Supplementary file 7 [file Image6.tif]

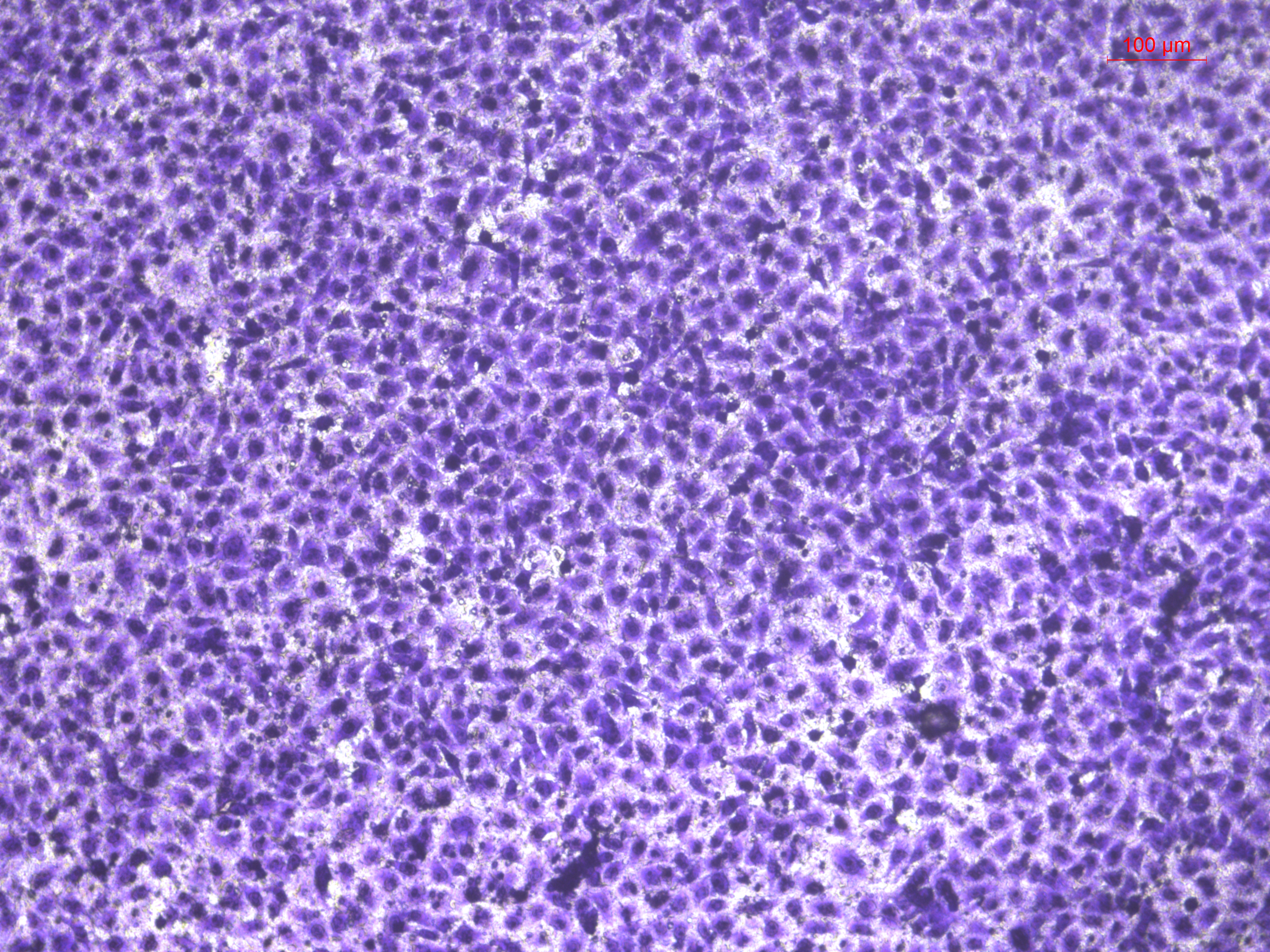

Supplement: Supplementary file 8 [file Image7.tif]

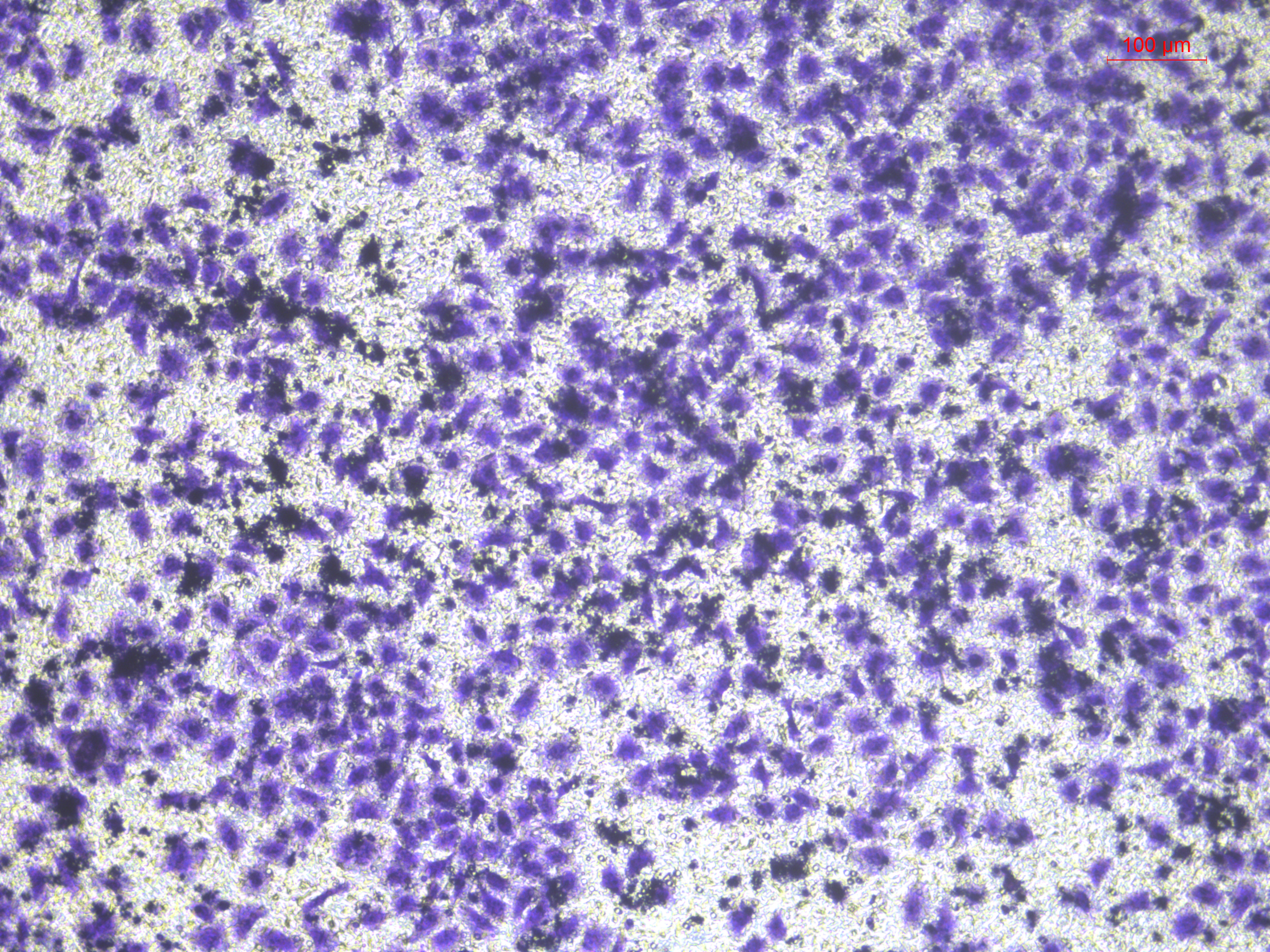

Supplement: Supplementary file 9 [file Image8.tif]
